# Associations between digital media use and brain surface structural measures in preschool-aged children

**DOI:** 10.1038/s41598-022-20922-0

**Published:** 2022-11-09

**Authors:** John S. Hutton, Jonathan Dudley, Thomas DeWitt, Tzipi Horowitz-Kraus

**Affiliations:** 1 Division of General and Community Pediatrics, Cincinnati Children’s Hospital Medical Center, University of Cincinnati College of Medicine, 3333 Burnet Avenue, MLC 2011, Cincinnati, OH 45229 USA; 2 Reading and Literacy Discovery Center, Cincinnati Children’s Hospital Medical Center, University of Cincinnati College of Medicine, Cincinnati, OH USA; 3 Pediatric Neuroimaging Research Consortium, Cincinnati Children’s Hospital Medical Center, University of Cincinnati College of Medicine, Cincinnati, OH USA; 4 Educational Neuroimaging Group, Education in Science and Technology, Biomedical Engineering, Technion, Haifa, Israel; 5 Kennedy Krieger Institute, Baltimore, MD USA; 6 Department of Psychiatry, Johns Hopkins University School of Medicine, Baltimore, MD USA

**Keywords:** Development of the nervous system, Paediatrics, Risk factors

## Abstract

The American Academy of Pediatrics recommends limits on digital media use (“screen time”), citing cognitive-behavioral risks. Media use in early childhood is ubiquitous, though few imaging-based studies have been conducted to quantify impacts on brain development. Cortical morphology changes dynamically from infancy through adulthood and is associated with cognitive-behavioral abilities. The current study involved 52 children who completed MRI and cognitive testing at a single visit. The MRI protocol included a high-resolution T1-weighted anatomical scan. The child’s parent completed the ScreenQ composite measure of media use. MRI measures included cortical thickness (CT) and sulcal depth (SD) across the cerebrum. ScreenQ was applied as a predictor of CT and SD first in whole-brain regression analyses and then for regions of interest (ROIs) identified in a prior study of screen time involving adolescents, controlling for sex, age and maternal education. Higher ScreenQ scores were correlated with lower CT in right-lateralized occipital, parietal, temporal and fusiform areas, and also lower SD in right-lateralized inferior temporal/fusiform areas, with substantially greater statistical significance in ROI-based analyses. These areas support primary visual and higher-order processing and align with prior findings in adolescents. While differences in visual areas likely reflect maturation, those in higher-order areas may suggest under-development, though further studies are needed.

## Introduction

The American Academy of Pediatrics (AAP) recommends limits on digital media use (“screen time”) for children at all ages^1^. Domains include access to screens, frequency of use, content and grownup-child co-viewing^1^. Cited risks of excessive and/or inappropriate use span developmental domains, including physical (e.g., obesity^2^), social-emotional (parent–child engagement^3^) and cognitive (e.g., language^4^, executive function^5,6^). Recent evidence suggests potential impacts on brain structure and function underlying these abilities^7–11^. Proposed mechanisms are direct (e.g. age-inappropriate content^12–14^, impaired sleep^15,16^) and indirect (e.g. displacement of parent–child interaction^10,17–20^) in nature. Despite these risks and recommendations, use has been increasing beginning in infancy, fueled by portable devices and amplified during the COVID-19 pandemic^21^.

Magnetic Resonance Imaging (MRI) is a powerful tool that can provide insights into relationships between environmental factors and brain structure and function. Several studies have explored neurobiological impacts of adverse childhood experiences, such as neglect and poverty^22–24^. However, few have explored relationships between digital media use and brain development, particularly during early childhood when plasticity is high. Higher media use referenced to AAP guidelines^1^ (ScreenQ measure^25^) was recently associated with lower microstructural integrity of major white matter tracts, and also with lower emergent literacy skills^26^. By contrast, other studies have found positive associations between shared reading at home and these white matter measures (and also functional MRI measures) at this age^27–29^, suggesting a potential displacement effect of screen use.

Early childhood (newborn through age 5) is a formative span of brain development^30,31^. Essential structural and functional networks are established by age two and then shaped by genetic and environmental factors, manifest via shifts in grey matter density (e.g., pruning, synaptogenesis) and myelination of white-matter tracts^30^. Cerebral surface morphology evolves across childhood, reflected by features such as cortical thickness (CT) and sulcal depth (SD). While developmental changes are non-linear and non-uniform, early childhood is an accretive stage of gray matter growth (i.e., thickening, deepening) with CT in most areas maximal by age 3 and SD maximal by late childhood^32–34^. However, maturation in limbic and sensory areas precedes that in higher-order areas (e.g., association, executive), which do not reach local maxima until adolescence^35^. Further, while thinning in sensory areas is thought to reflect maturation, greater CT and SD in higher-order areas have been linked to a range of cognitive abilities in children, adolescents and young adults^36–42^. While there have been few such studies involving preschool-age children, higher CT in occipital-parietal-temporal areas known to support reading were recently associated with higher language and emergent literacy skills^43^.

A recent analysis from the large, ongoing Adolescent Brain Cognitive Development (ABCD) study found associations between higher digital media use (reported minutes/day) and lower CT and SD in areas involved with visual processing, executive functions, memory and attention^7^. The authors attributed findings to accelerated maturation of the visual system, yet noted thinning in areas that are not functionally homologous, suggesting non-uniform impacts of media use that are less clear. Potential correlates included higher externalizing behaviors for children with higher use.

The objective of the current study was to explore relationships between reported digital media use and measures of CT and SD in a sample of healthy preschool-age children during a rapid span of bran development. While relatively little is known at this age, the hypotheses were that higher use would be associated with lower CT and SD in, (1) occipital areas, reflecting accelerated maturation of the visual system expected to be in a reductive phase at this age, and (2) frontal-parietal-temporal areas, reflecting relative under-development of higher-order areas expected to be in an accretive phase at this age. To address concerns about limited statistical power for this moderate sample size and to account for demographic covariates, analyses included a regions of interest (ROIs) approach limited to areas where differences in CT and SD were most strongly associated with digital media use in the ABCD study^7^.

## Material and methods

### Overview/design

The current study is a secondary analysis of data collected for an MRI-based study involving impacts of home reading practices and digital media use on brain structure and function supporting emergent literacy skills in preschool-age children^26,44^.

### Screen time measure (ScreenQ)

The ScreenQ is a 15-item parent-report measure of digital media use developed by the study team^45^. Its conceptual model involves four domains featured in AAP recommendations for young children: access to screens, frequency of use, content and parent–child co-viewing^1^. Internal consistency (Cronbach α = 0.74), reliability and concurrent validity have been established in young children and more recently via wider age range using a Portuguese translation^25,46^.

### Participants/setting

Healthy children between 3- and 5-years old were recruited at a pediatric academic center and primary care clinics in a large Midwestern city. Eligibility criteria were: (1) gestation ≥ 36 weeks, (2) age 36–52 months, (3) no prior or current kindergarten attendance, (4) no documented history of head trauma with loss of consciousness or neurodevelopmental condition likely to confer cognitive delay, (5) native English-speaking custodial parent, and (6) no contraindications for MRI such as metal implants, orthodontic braces or claustrophobia. Written informed consent was obtained from a parent and families were provided with financial compensation for time and travel.

This study was approved by the Cincinnati Children’s Hospital Institutional Review Board. All research was performed in accordance with human subjects protections guidelines in accordance with the Declaration of Helsinki principles.

### Screening and assessments

Clinical research coordinators collected demographic information and administered the ScreenQ to the child’s parent in a private room before the MRI scan. Standard measures of expressive language (Expressive Vocabulary Test, 2nd Edition; EVT-2), processing speed (Comprehensive Test of Phonological Processing, rapid object naming subscale; CTOPP-2), rhyming abilities (Pre-reading Inventory of Phonological Awareness, rhyming subscale; PIPA) and emergent literacy composite (Get Ready to Read; GRTR^47^) skills were administered to the child prior to MRI, and have been reported previously^26,44^.

### Descriptive analyses

Descriptive statistics were computed for demographic and other variables featured here, specified in a statistical analysis plan. Poverty status was defined using 2020 US Department of Health and Human Services criteria, using the midpoint of income category relative to household size^48^. Analyses were conducted using SAS v9.4 software.

### Magnetic resonance imaging (MRI)

Details of play-based acclimatization techniques prior to MRI have been described previously^49^. The protocol involved structural and functional MRI, but only the T1-weighted structural scan was used for the current study. Children were awake and non-sedated during MRI, which was conducted using a 3-Tesla Philips Ingenia scanner with a 32-channel head coil. High-resolution, 3D T1-weighted anatomical images were acquired (TR/TE = 8.1/3.7 ms; duration 5.25 min; FOV = 256 × 256 mm; matrix = 256 × 256; in-plane resolution = 1 × 1 mm; slice thickness = 1 mm; number of slices = 180, sagittal plane). Processing utilized the Computational Anatomy Toolbox (CAT12, Structural Brain Mapping Group, Jena, Germany), which performs non-linear transformations for voxel-based preprocessing, then computes surface-based morphometric (cortical thickness) measures. Individual subjects were mapped to a standard template space (~ 2 mm spacing) using age-matched a prior tissue probability maps generated from the TOM8 toolbox^50^ for tissue segmentation. After this voxel-based spatial registration, the central surface and morphometric measures (CT, SD) were determined using the projection-based thickness method. The central surface was then spatially registered to the Freesurfer “FsAverage” template. Finally, measures of CT and SD were projected onto the template space and then smoothed along the surface with a 10 mm and 15 mm full-width half-maximum Gaussian kernel, respectively. Subjects with weighted image quality (calculated based on resolution, signal-to-noise ratio, and bias field strength) of 2 or more standard deviations below the group mean and/or subjects with a mean correlation coefficient of CT 2 standard deviations or more below the group mean were excluded as outliers.

### Regions of interest for MRI analyses

To increase statistical power, regions of interest (ROIs) were selected based on the largest effect sizes involving digital media use and CT and SD, respectively, in a recently published MRI study involving a large sample of young adolescents^7^. These were selected from group factor analyses 1 and 3 in that study, which loaded most strongly on overall digital media use, which was considered most similar to the ScreenQ measure, as opposed to specific usage factors such as social media. These were defined via the Desikan-Killiany cortical atlas^51^, as in the prior work. Given the young age of the subjects where many cortical functions are less likely to have lateralized (e.g., language), bilateral ROIs were included. For CT, the ROIs selected were bilateral cuneus, fusiform, inferior temporal, lateral occipital, lingual, pericalcarine, postcentral, precuneus, superior parietal and supramarginal gyri. For SD, the ROIs were bilateral cuneus, fusiform, inferior temporal, lateral occipital, lingual and pericalcarine gyri.

### MRI analyses

Analyses involved multiple regression modeling with CT and SD as the respective dependent variable, applying ScreenQ score (continuous) as the predictor and controlling for covariates sex (categorical), age (continuous) and maternal education level (categorical). Maternal education level was chosen as a proxy for socioeconomic status (SES), as it has been cited as most strongly associated with child cognitive and social-emotional development^52^. Smoothed thickness maps were fit to these models to estimate the effect of ScreenQ total scores on CT and SD across the cerebrum. These were then computed for the ROIs identified above in respective analyses of CT and SD, controlling for these covariates. To account for multiple comparisons testing, False Discovery Rate (FDR) correction was applied for all analyses using thresholds of α = 0.05 and also a more liberal α = 0.10, with a two-sided test.

## Results

### Sample characteristics and ScreenQ scores

A total of 58 children completed MRI, 52 of them with acceptable image quality for analyses, applying criteria described above (age 52.7 ± 7.7 months-old, range 37–63; 29 girls, 23 boys). The mean ScreenQ score for those included was 10.1 ± 4.5 (range 3–21). ScreenQ scores were negatively associated with maternal education level (Pearson r = − 0.41, *p* < 0.001).

These data are summarized in Table 1.

**Table 1:** Demographics and ScreenQ Scores.

Demographics and ScreenQ scores	N (%)	Mean ± SD (Min, Max)
Total	52 (100)	52.7 ± 7.7 (37, 63)
**Child Age (months)**		
36+	15 (29)	
48+	23 (44)	
60+	14 (27)	
**Child gender**		
Male	23 (44)	
Female	29 (56)	
**Annual household income ($)**		
≤ 25,000	7 (13)	
25,001–50,000	9 (17)	
50,001–100,000	15 (29)	
100,001–150,000	11 (21)	
Above 150,000	10 (19)	
*Income Relative to Needs		
At or under poverty threshold	9 (17)	
Above poverty threshold	43 (83)	
**Maternal Education**		
High School or Less	4 (8)	
Some College	9 (17)	
College graduate	22 (42)	
More than college	17 (33)	
ScreenQ total score	52 (100)	10.1 ± 4.5 (3, 21)

*2020 US Department of Health and Human Services Poverty Table (income to household size).

### Cognitive-behavioral analyses

In previously published studies involving this cohort, higher ScreenQ scores were associated with significantly lower expressive language (EVT-2, scaled), processing speed (CTOPP-Rapid Object Naming, scaled), rhyming (PIPA, scaled) and emergent literacy composite (GRTR, total) scores (all *p* < 0.05)^26,44^.

### MRI analyses

**Figure 1:**
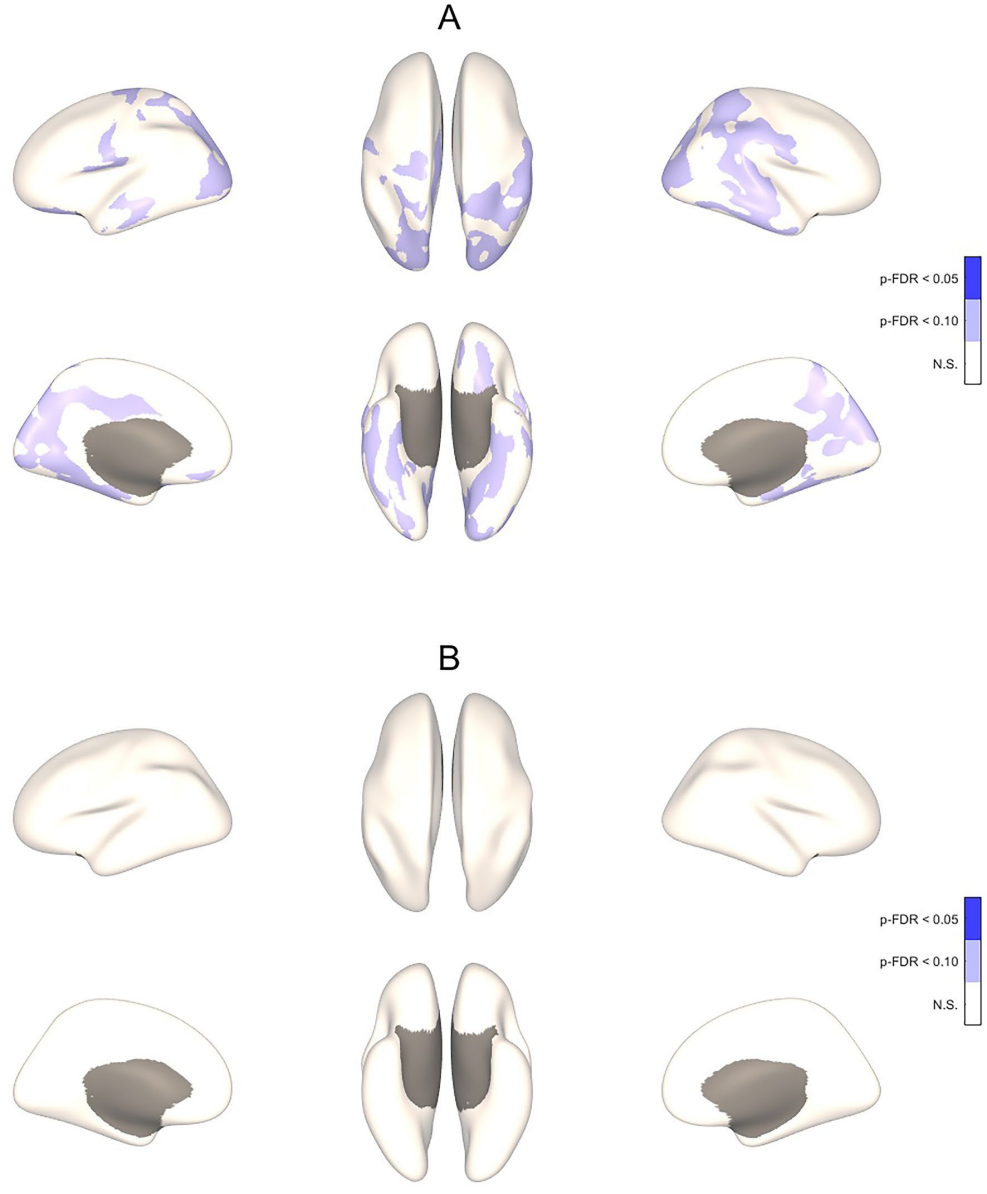
3-D Maps Showing Correlation Between ScreenQ Scores and Cortical Thickness for the Whole Brain. Three-dimensional maps showing correlations between *ScreenQ* total scores and cortical thickness for the whole cerebrum, controlling for age and sex (**A**) and also for maternal education (**B**). These are displayed on an inflated brain for better visibility of clusters, with blue representing thinner cortex. Upper views are lateral and superior, lower views are medial and inferior, with the frontal lobe facing upward. Cortical regions surviving p-FDR < 0.10 and shown in (**A**) are detailed in Table 2.

**Figure 2:**
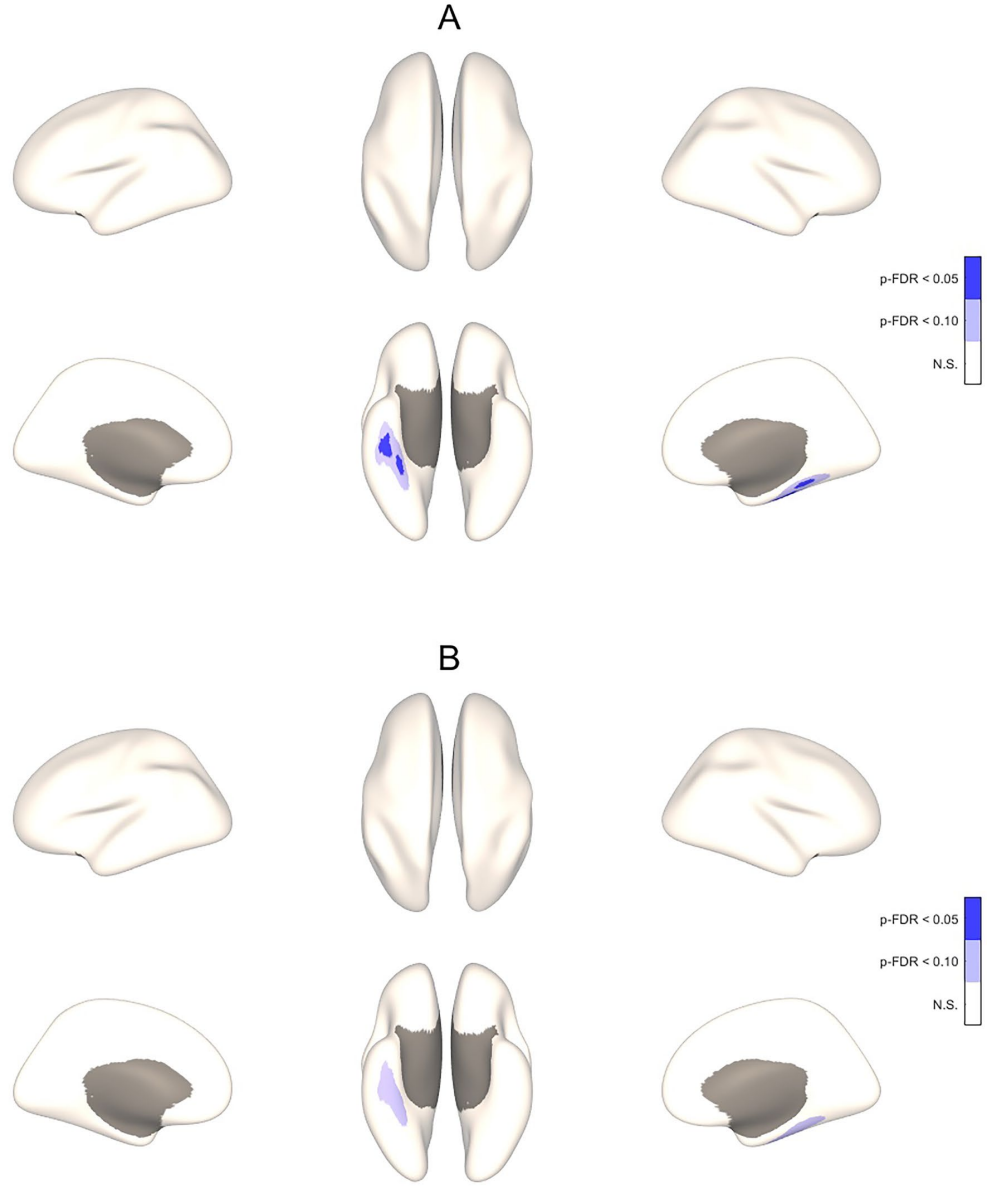
3-D Maps Showing Correlation Between ScreenQ Scores and Sulcal Depth for the Whole Brain. Three-dimensional maps showing correlations between *ScreenQ* total scores and sulcal depth for the whole cerebrum, controlling for age and sex (**A**) and also for maternal education (**B**). These are displayed on an inflated brain for better visibility of clusters, with blue representing shallower depth. Upper views are lateral and superior, lower views are medial and inferior, with the frontal lobe facing upward. Numbered cortical regions surviving p-FDR < 0.05 and p-FDR < 0.10 and shown in (**A**) and (**B**) are detailed in Table 3.

In whole-brain analyses, higher ScreenQ scores were correlated with lower CT in extensive clusters located in bilateral yet right-lateralized occipital, parietal, temporal and fusiform regions, controlling for sex and age, though with marginal statistical significance (two-tailed p-FDR < 0.10), shown in Fig. 1A and detailed in Table 2. When adding maternal education (SES) as an additional covariate, these associations did not reach statistical significance (Fig. 1B, Table 2). Higher ScreenQ scores were also correlated with lower SD in two clusters located in the right fusiform cortex, controlling for sex and age (two-tailed p-FDR < 0.05), shown in Fig. 1B and detailed in Table 3. When adding maternal education (SES) as a covariate, the extent of these associations was similar yet with marginal statistical significance (p-FDR < 0.10), shown in Fig. 2B and summarized in Table 3.

**Table 2:** Details of significant clusters from Fig. 1.

Cluster #	p-FDR	MNI coordinates	Regions	Major function
1	0.089	55 − 19 − 34	69% Postcentral 31% Supramarginal	Somatosensory, emotional processing Social cognition, proprioception
2	0.081	29 − 51 − 43	77% Superior Parietal 23% Inferior Parietal	Focused attention Multisensory association, emotional processing, music, math operations
3	0.076	29 − 85 − 4	98% Lateral Occipital 2% Inferior Parietal	Primary visual Multisensory association, emotional processing, music, math operations
4	0.097	25 − 38 − 54	55% Postcentral 45% Superior Parietal	Somatosensory, emotional processing Focused attention
5	0.099	45 − 37 − 42	100% Supramarginal	Social cognition, proprioception

Corrected *p*-value, location, atlas labels and major function of clusters with lower cortical thickness (CT) correlated with higher ScreenQ scores controlling for child sex and age, shown in Fig. 1A (thresholds: two-sided p-FDR < 0.10 and p-FDR < 0.05). Montreal Neurological Institute (MNI) coordinates are left–right, posterior-anterior and inferior-superior relative to the anterior commissure. Regions indicates the percentage of each cluster residing in the respective Desikan-Killiany DK40 atlas-defined area.

**Table 3:** Details of significant clusters from Fig. 2

Cluster #	p-FDR	MNI coordinates	Regions	Major function
1 (2A)	0.046	42 − 19 − 23	66% Fusiform 34% Inferior Temporal	Visual processing (shapes, letter/word forms), imagery, semantic memory and retrieval Visual processing, emotional regulation
2 (2A)	0.049	35 − 39 − 16	98% Fusiform 2% Parahippocampus	Visual processing (shapes, letter/word forms), imagery, semantic memory and retrieval Emotional learning, memory
3 (2B)	0.071	42 − 19 − 23	74% Fusiform 21% Inferior Temporal 5% Parahippocampus	Visual processing (shapes, letter/word forms), imagery, semantic memory and retrieval Visual processing, emotional regulation Emotional learning, memory

Corrected *p*-value, location, atlas labels and major function of clusters with lower sulcal depth (SD) correlated with higher ScreenQ scores controlling for child sex and age, shown in Fig. 2A and also controlling for maternal education shown in Fig. 2B (thresholds: two-sided p-FDR < 0.05 for 2A and p-FDR < 0.10 for 2B). MNI coordinates are left–right, posterior-anterior and inferior-superior relative to the anterior commissure. Regions indicates the percentage of each cluster residing in the respective Desikan-Killiany DK40 atlas-defined area.

**Figure 3:**
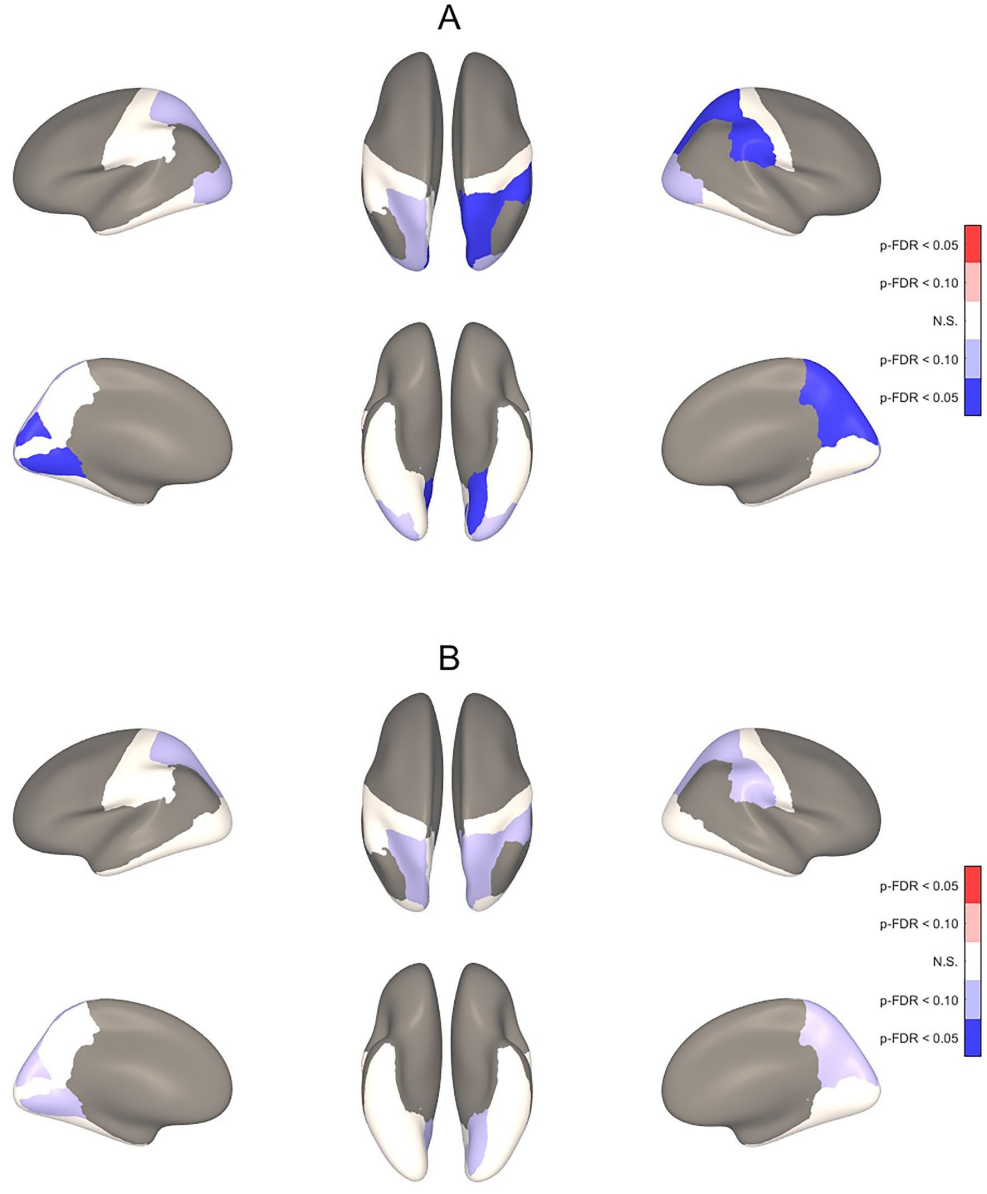
3-D Maps Showing Correlation Between ScreenQ Scores and Cortical Thickness for Defined Regions of Interest. Three-dimensional maps showing correlations between *ScreenQ* total scores and cortical thickness for defined regions of interest (ROIs), controlling for age and sex (**A**) and also for maternal education (**B**). These are displayed on an inflated brain for better visibility of clusters, with blue representing thinner cortex. Upper views are lateral and superior, lower views are medial and inferior, with the frontal lobe facing upward. Effect sizes and FDR-corrected p-values for these ROIs in (**A**) and (**B**) are detailed in Table 4.

**Figure 4:**
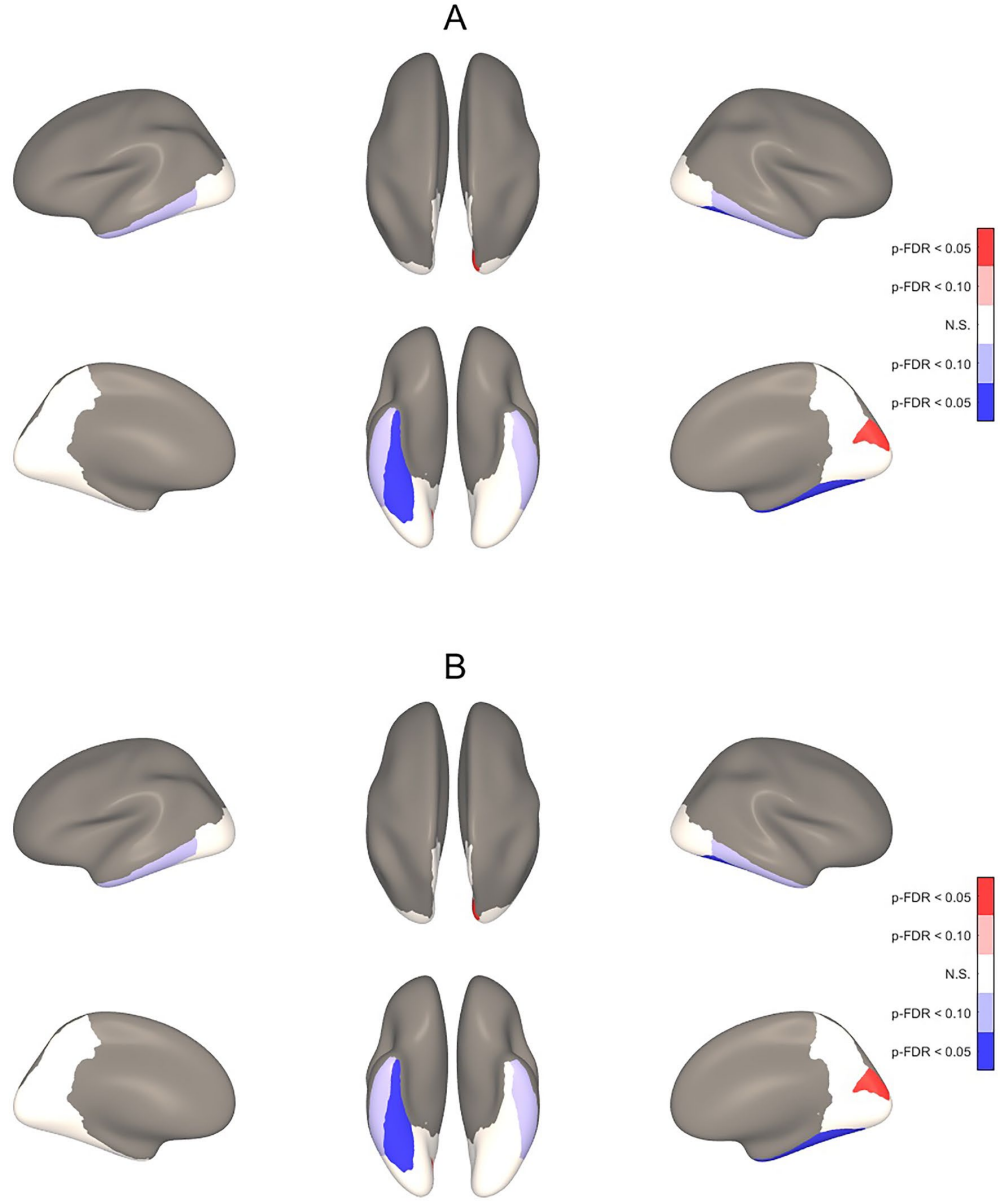
3-D Maps Showing Correlation Between ScreenQ Scores and Sulcal Depth for Defined Regions of Interest. Three-dimensional maps showing correlations between *ScreenQ* total scores and sulcal depth for defined regions of interest (ROIs), controlling for age and sex (**A**) and also for maternal education (**B**). These are displayed on an inflated brain for better visibility of clusters, with blue representing shallower and red representing deeper. Upper views are lateral and superior, lower views are medial and inferior, with the frontal lobe facing upward. Effect sizes and FDR-corrected p-values for these ROIs in (**A**) and (**B**) are detailed in Table 5.

For the ROI-based analyses, higher ScreenQ scores were correlated with lower CT in bilateral cuneus, left lingual gyrus and right precuneus, superior parietal and supramarginal gyri, controlling for sex and age (two-tailed p-FDR < 0.05), shown in Fig. 3A and detailed in Table 4. When applying material education (SES) as an additional covariate, the extent of associations was similar, yet with marginal statistical significance (two-tailed p-FDR < 0.10), shown in Fig. 3B and detailed in Table 4. Higher ScreenQ scores were also correlated with greater SD in the right cuneus and lesser SD in the right fusiform gyrus (two-tailed p-FDR < 0.05) and marginally lesser SD in the left inferior temporal gyrus, shown in Fig. 4A and detailed in Table 5. When adding maternal education (SES) as an additional covariate, the extent of associations was nearly identical and remained statistically significant at p-FDR < 0.05 for the cuneus and fusiform areas, shown in Fig. 4B and detailed in Table 5.

**Table 4:** Cohen’s d effect sizes and false-discovery rate (FDR) corrected p-values for associations between ScreenQ and cortical thickness for selected regions of interest (ROIs) defined by the Desikan-Killiany cortical atlas and shown in Fig. 3.

ROI	Left hemisphere			Right hemisphere		
	Effect size	p-FDR	p-FDR_SES_	Effect size	p-FDR	p-FDR_SES_
Cuneus C	− 0.65	0.042*	0.085	− 0.68	0.042*	0.085
Fusiform G	− 0.33	0.131	0.175	− 0.31	0.131	0.175
Inferior Temporal G	0.04	0.433	0.449	− 0.48	0.107	0.123
Lateral Occipital C	− 0.44	0.062	0.131	− 0.45	0.075	0.131
Lingual G	− 0.72	0.040*	0.085	− 0.07	0.304	0.434
Pericalcarine C	− 0.32	0.122	0.175	− 0.19	0.220	0.288
Postcentral G	− 0.31	0.131	0.175	− 0.32	0.122	0.175
Precuneus C	− 0.36	0.122	0.175	− 0.58	0.042*	0.085
Superior Parietal C	− 0.55	0.056	0.090	− 0.59	0.042*	0.085
Supramarginal G	− 0.01	0.374	0.483	− 0.58	0.042*	0.085

Negative signs added to effect sizes indicate a negative association between ScreenQ score and cortical thickness.

*p-FDR *false-discovery rate corrected *p*-value controlling for age and sex, * p-FDR*_*SES*_ false-discovery rate corrected *p*-value controlling for age, sex and socioeconomic status (maternal education), *C *cortex, *G *gyrus.

*Signifies that p-FDR is less than 0.05, defined as statistically significant (p-FDR < 0.10 is defined as marginally statistically significant).

**Table 5:** Cohen’s d effect sizes and false-discovery rate (FDR) corrected p-values for associations between ScreenQ and sulcal depth for selected regions of interest (ROIs) defined by the Desikan-Killiany cortical atlas and shown in Fig. 4.

ROI	Left hemisphere			Right hemisphere		
	Effect size	p-FDR	p-FDR_SES_	Effect size	p-FDR	p-FDR_SES_
Cuneus C	0.46	0.283	0.184	0.80	0.032*	0.026*
Fusiform G	− 0.10	0.442	0.485	− 0.80	0.045*	0.035*
Inferior Temporal G	− 0.60	0.091	0.084	− 0.56	0.091	0.084
Lateral Occipital C	0.00	0.442	0.500	− 0.27	0.186	0.294
Lingual G	0.17	0.299	0.421	− 0.27	0.442	0.294
Pericalcarine C	0.11	0.445	0.421	− 0.03	0.442	0.500
Precuneus C	0.06	0.445	0.425	0.33	0.299	0.261

Negative signs added to effect sizes indicate a negative association between ScreenQ score and sulcal depth.

*p-FDR *false-discovery rate corrected *p*-value controlling for age and sex,* p-FDR*_*SES*_ false-discovery rate corrected *p*-value controlling for age, sex and socioeconomic status, *C *cortex, *G *gyrus.

*Signifies that p-FDR is less than 0.05, defined as statistically significant (p-FDR < 0.10 is defined as marginally statistically significant).

## Discussion

Brain development is a dynamic, non-linear process influenced by genetic and environmental factors. Environmental influences include relationships and experiences and can be nurturing, adverse or neutral. Given the prominent and increasing role of digital media for families beginning in infancy, it is critical to understand the direct and indirect impacts of various aspects of use on emerging skills and underlying neurobiology. These are likely to be greatest during early childhood when brain networks develop rapidly and plasticity is high, manifest via differences in gray and white matter structure^30^. However, currently, very little is known about these potential impacts. The purpose of this study was to examine associations between digital media use and established measures of cortical morphology (CT, SD) at this formative age. In line with our hypotheses, in both whole-brain and ROI-based analyses, higher media use was related to differences in CT (all lesser) and SD (primary visual greater, higher-order lesser) in both primary visual and higher-order association areas.

Cortical thickness (CT) reflects synaptic density and supporting cellular architecture^53^. While overall CT reaches maximal levels by age 2, that of limbic and sensory areas precedes higher-order (e.g. association, executive) areas, which do not achieve local maxima until adolescence^35^. It has been suggested that thickness may even be a marker for “lower” sensory processes (thinning occurs earlier) versus “higher” associative and integrative processes (thinning occurs later)^54^. Changes reflect cortical remodeling in response to environmental stimulation, which can be accretive (e.g., synaptogenesis) or reductive (e.g., pruning)^53^. The current study involved 3–5-year old children, whose overall CT is expected to have largely peaked, though not yet in higher-order areas. Despite limited statistical power, particularly when controlling for maternal education, significant (ROI-based) and/or marginally significant (whole-brain) associations were identified between higher screen-based media use and lesser CT involving both primary and higher-order areas. The most extensive and significant clusters were in right-lateralized occipital and superior parietal regions (Figs. 1 and 3) that support both sensory (e.g., cuneus) and multi-modal associative (e.g., supramarginal gyrus) processes, suggesting impacts in areas expected to be mature at this age and in others that are expected to still be developing.

Synchronous thinning in functionally related areas has been linked to environmental factors (e.g., visual network via visual stimuli)^42^. Thinning in visual cortices has also been attributed to higher maturation and efficiency^7^. Association between higher ScreenQ scores and lower CT in bilateral, right-lateralized occipital areas (cuneus) in the current study is consistent with these models, possibly via greater exposure to screen-based media during early childhood. Higher ScreenQ scores were also associated with lower CT in the right superior parietal lobe, which is a major node in the “top-down” dorsal attention network, particularly involving visual-spatial stimuli^55^. Whether this finding reflects accelerated maturation via more frequent and/or stimulating screen-based media use, or under-development via less exposure to non-screen stimuli (e.g., shared reading) is unclear and in need of further study.

By contrast to primary visual areas, lower CT in the lingual gyrus, which is considered to be a higher-order visual-association area, was left-lateralized (especially ROI-based, Fig. 3), suggesting asynchronous thinning that tends to occur in these specialized brain areas. Adjacent to the parahippocampus, the lingual gyrus is involved with complex visual memory encoding, including facial and emotional expressions, core social-cognitive processes^56^. Lower CT in the lingual gyrus has been linked to lower episodic memory and social cognition in adults^57^. The lingual gyrus has also been found to support printed letter recognition, a pre-reading skill that typically develops in the preschool-age range, with greater left-lateralization linked to higher skill^58,59^. As both social cognition and emergent literacy skills are typically in early stages of development in the formative preschool age range, lower CT found here may reflect under-development rather than efficiency, though this is speculative and in need of further study.

Association between higher ScreenQ scores and lower CT in the postcentral gyrus, whose major role is somatosensory processing, is more counter-intuitive. A reasonable potential mechanism involves the stimulation of mirror neurons during the processing of imagined sensations in video scenes^60,61^. Indeed, these clusters with lower CT were in the more posterior Brodmann Area 2, where mirror neurons are well-documented^62^ and which supports higher-order somatosensory processing and social cognition^63^. Thus, if this mechanism is accurate, a major question is whether somatosensory cortical remodeling via digitally presented scenes is of functional relevance compared to thinning that may manifest via real-world human-interactive situations.

In contrast to primary sensory areas where thinning is generally adaptive, CT in higher-order areas (e.g., executive, association) has been positively associated with cognitive performance, including IQ, language, social cognition and emergent literacy skills^36–38,64,65^. Thus, akin to findings involving the lingual gyrus, it is less clear whether associations between higher ScreenQ scores and lower CT in the right inferior parietal lobe, which supports multi-modal (e.g., visual, somatosensory, emotional) processing^66^ and also learned and creative skills such as music^67^ and math^68^, are benign or maladaptive in nature. Similarly, higher media use was associated with lower CT in the right supramarginal gyrus (SMG), a higher-order area not expected to have peaked at preschool age. The right SMG supports empathy (in children, overcoming egocentricity bias)^69,70^, and lower CT in this area has been linked to conduct disorder in adolescents^71^. While not assessed here, excessive and inappropriate digital media use has been linked to lower empathy^72^, and a “video-deficit” in social cognition described in preschool-age children^73^. Thus, while speculative, findings in the current study may reflect SMG under-development at this age, an additional potential early biomarker of impacts of higher media use on social cognition. Interestingly, the postcentral gyrus is also involved with emotional processing and empathy (largely via the mirror neuron system), with lower CT possibly suggesting maladaptive neurodevelopment in these domains^63^. Further studies involving measures of social cognition are needed to better characterize these potential impacts.

The current findings align with those from the large, ongoing "ABCD" study involving early-adolescent children, where higher media use was associated with lower CT in both sensory (e.g., primary visual, postcentral) and higher-order (e.g., fusiform, SMG) areas^7^. The authors attributed these findings to accelerated maturation of the visual system, with impacts on other, non-functionally homologous areas less clear. At a minimum, findings in the current study involving visual areas are consistent with those in the ABCD study, suggesting that relationships between higher media use and brain structure begin to manifest in early childhood and may become more extensive over time. They are also consistent with recent functional MRI studies involving preschool-age children presented with stories in illustrated and animated formats, where functional connectivity involving primary visual networks was substantially higher during the animated story, a potential mechanism for accelerated thinning^74,75^.

Sulcal depth (SD) is an established measure of cortical surface area, which exhibits more gradual maturational changes with age, reaching overall maxima in late childhood^35,53,76^. The current study found significant association between higher ScreenQ scores and significantly greater SD in primary visual cortex (right cuneus), which may reflect accelerated maturation in concert with lower CT. By contrast, higher ScreenQ scores were associated with significantly lesser SD in the right fusiform gyrus, which supports higher-order processing of complex visual stimuli (e.g., faces, places, shapes)^77,78^. The fusiform cortex also includes the putative Visual Word Form Area (VWFA), which gradually develops to rapidly process letters and words during reading^79^. Greater SD (and also CT) in the fusiform cortex has been associated with higher reading abilities^41,80^, including at young ages before formal reading instruction^81^ and with higher emergent literacy skills^43^. They also align with associations between higher media use (ScreenQ) and both lower emergent literacy skills and white matter microstructural integrity supporting these skills found in a related study involving preschool-age children^26^. Thus, while speculative, the current findings may be a biomarker of impacts of higher screen-based media use on cortical surface area (SD) supporting reading at this age, though further studies are needed.

This study has limitations that should be noted. While 17% of participants met poverty criteria, the sample was largely of higher income and maternal education, and results might be different with greater socioeconomic diversity. There were few significant findings applying maternal education level as a covariate alongside child age and sex, attributable to limited statistical power and moderate correlation between this covariate and ScreenQ scores, which is consistent with prior studies linking media use to numerous aspects of SES^82^. However, these analyses still generated significant and/or marginally significant results aligned with previous studies involving early adolescents^7^, to inform more expansive research. Analyses were limited to children completing MRI and meeting necessary motion criteria, which may bias results towards those with higher self-regulation and other behavioral characteristics. The cross-sectional nature prohibits comment on causality, which requires a longitudinal design. It is also impossible to discern whether associations between higher media use and differences in CT and SD stemmed from direct (e.g., visual stimulation) or indirect (e.g., displacement of reading) mechanisms. While differences in cortical morphology related to higher use were found at a single time point, rates of change may be more relevant to cognitive development^83^. Finally, while there were structural differences in areas known to support higher-order skills (e.g., social cognition, emergent literacy), only measures related to emergent literacy were administered (all negatively correlated, reported previously)^26,44^, rendering brain-behavior relationships speculative. Future studies incorporating a range of cognitive-behavioral measures at this formative age are needed.

This study also has important strengths. It involves a reasonably large sample of very young children, where there have been few MRI-based studies involving media use, and none to our knowledge involving cortical structure. Rather than a single aspect of use, it applies ScreenQ as its predictor variable, which is a validated, composite measure^25,46^ capturing evidence-based facets of use cited in AAP recommendations^1^. Analyses involved CT and SD, complimentary measures with non-uniform developmental trajectories, reflecting synapse-level changes and brain growth^35^. All controlled for age and sex, minimizing the influence of general maturation rather than environment^34,84,85^. While impacting statistical power, significant and/or marginally significant results were found controlling for maternal education, which has been cited as a major SES-related predictor of child cognitive and social-emotional development^52^. All analyses applied conservative false-discovery rate (FDR) correction, reducing the likelihood of false positive results. Perhaps most importantly, the current findings align with those involving CD and SD in the large ABCD study involving older children^7^, and complement previous studies at this age involving differences in cognitive skills, functional connectivity and white matter microstructure^26,74,75^.

Altogether, while several findings are unclear and/or speculative, attributable to the complex nature of cortical development, this study provides novel evidence that differences in brain structure related to screen-based media use are evident during early childhood. Longitudinal studies, ideally beginning in infancy given trends in digital media use and prevalence of portable devices^86,87^, are needed to characterize longer-term impacts on cognitive, social-emotional and overall health outcomes.

## Conclusions

This study found associations between higher digital media use and lower cortical thickness and sulcal depth in brain areas supporting primary visual processing and higher-order functions such as top-down attention, complex memory encoding, letter recognition and social cognition. These findings are consistent with those from a large study involving adolescents, suggesting that differences in cortical structure related to screen use may begin to manifest in early childhood. They also compliment associations between higher media use and lower cognitive skills and related white matter microstructure previously found at this age. Further studies are needed to determine the longer-term evolution and relevance of these structural differences in terms of cognitive, social-emotional and overall development.

## Data Availability

All survey and MRI data for this study were newly acquired via methods described. These data will be made available to the scientific community in a deidentified manner upon notice of publication via written request to the corresponding author (JH). Requests must include description of the project (e.g., project outline) and also acknowledgment of the data source in any grant submissions, presentations or publications. The rationale for written request is that no repository currently exists and creation would exceed the scope and current funding resources of the study team. Any costs associated with data transfer will be the responsibility of the requesting parties. Software utilized in the current analyses is freely available and described in the methods section.

## Abbreviations

AAP: American academy of pediatrics
FWE: Family-wise error
MNI: Montreal neurological institute
MRI: Magnetic resonance imaging
SES: Socioeconomic status
